# The application basis of immuno-checkpoint inhibitors combined with chemotherapy in cancer treatment

**DOI:** 10.3389/fimmu.2022.1088886

**Published:** 2023-01-10

**Authors:** Ming-Yan Shi, Han-Ge Liu, Xiao-Hong Chen, Ye Tian, Zhi-Nan Chen, Ke Wang

**Affiliations:** National Translational Science Center for Molecular Medicine & Department of Cell Biology, Fourth Military Medical University, Xi’an, China

**Keywords:** immuno-checkpoint inhibitors, chemotherapy, tumor microenvironment, immunogenic cell death, anti-tumor activity

## Abstract

Immuno-checkpoint inhibitors (ICIs) bring a promising prospect for patients with cancers, which restrains the growth of tumor cells by enhancing anti-tumor activity. Nevertheless, not all patients benefit from the administration of ICIs monotherapy. The partial response or resistance to ICIs is mainly due to the complex and heterogenous tumor microenvironment (TME). The combined therapy is necessary for improving the efficacy of tumor treatment. Chemotherapy is reported not only to kill tumor cells directly, but also to stimulate effective anti-tumor immune responses. Several combined therapies of ICIs and chemotherapeutic agents have been approved for the first-line treatment of cancers, including PD-1/PD-L1 inhibitors. This review summarizes the potential mechanisms of the combined therapy of ICIs and chemotherapeutic agents in inducing immunogenic cell death (ICD) and reprogramming TME, and elucidates the possible anti-tumor effects of combined therapy from the perspective of metabolic reprogramming and microbiome reprogramming.

## Introduction

Malignant tumors, as a type of incurable diseases, have threatened human health seriously owing to the immunosuppressive tumor microenvironment. The tumor immunotherapies dramatically make a monumental breakthrough for cancer treatment and bring significant improvement for patient survival by boosting effective immune response to eliminate malignant cells (1, 2). Oncolytic virus therapies, cancer vaccines, cytokine therapies, adoptive cell transfer therapies, and ICIs are included, and ICIs, which include inhibitors of the programmed cell death protein 1 (PD-1), programmed cell death-ligand 1 (PD-L1), and the cytotoxic T lymphocyte-associated protein 4 (CTLA-4), have been broadly used in clinical applications and contribute to prolonged survival for lung cancer patients (2–5).

However, not all patients benefit from immune checkpoint blockade therapy (6, 7). Based on response to ICIs, three broad population of patients are identified, which include responders, those that acquire resistance, and those that never respond (8–10). Unfortunately, only 20% of NSCLC patients response to ICIs, which shows a relatively lower clinical effect compared to other cancers (7). The complex and heterogenous TME is reported to be involved in the response to ICIs (11, 12). Based on the status of T cell infiltration, TME is classified as the immune-inflamed phenotype, the immune-excluded phenotype, and the immune-desert phenotype (13, 14). The reactivation and clonal-proliferation of antigen-experienced T cells in the TME are necessary for an effective anti-tumor response after ICI administration. Nevertheless, the tumor-associated macrophages (TAMs), cancer-associated fibroblasts (CAFs), myeloid-derived suppressor cells (MDSCs), and regulated T cells (Tregs) in TME have an inhibitory impact on the infiltration and activation of effector T cells (15). As a result, ICIs combined with other therapies that activate the immune effects of TME seems to be a better choice for tumor treatment. As of December 2021, 4,897 clinical trials are conducted to test the efficacy of PD-1/PD-L1 inhibitors. Among them, 83% are ICIs combined with other therapies, such as chemotherapy, radiotherapy, and other immuno-oncology therapies (16). Several clinical trials show that ICIs combined with chemotherapy have a better clinical effect compared to ICIs monotherapy (17–19). For example, pembrolizumab, a humanized monoclonal antibody against PD-1, plus platinum-based chemotherapy significantly improved overall survival rates of NSCLC patients with a PD-L1 ≥ 50% and negative for genomic alterations in the EGFR and ALK genes, compared with pembrolizumab monotherapy (19). Meanwhile, ICIs combined with chemotherapy also prolong the NSCLC patient survival, compared with chemotherapy (20–23), which have been approved for the first-line treatment in advanced NSCLC patients (24).

**Figure 1:**
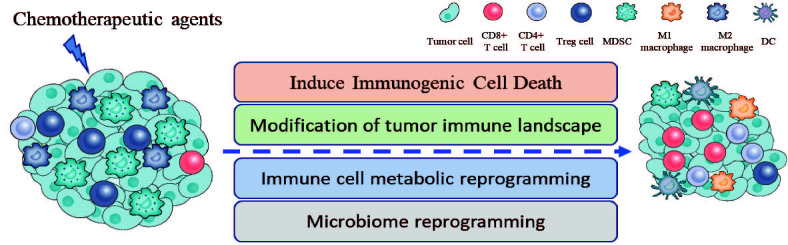
Chemotherapeutic agents enhance the anti-tumor activity of ICIs through several mechanisms, including inducing immunogenic cell death, changing the proportion and activity of immune cells in TME, immune cell metabolic reprogramming, and microbiome reprogramming.

In this review, we summarize the synergetic effects of chemotherapy with ICI therapy in tumor treatment from the perspective of inducing ICD, remodeling TME, metabolic reprogramming, and microbiome reprogramming (**Figure 1**). In addition, further researches need to be conducted to explore the novel mechanisms of above-mentioned therapy in cancers, which may provide a solid foundation for future clinical applications.

## Inducing immunogenic cell death

Several studies have shown that chemotherapeutic agents have the ability of inducing ICD in animal experiments, including anthracyclines, cyclophosphamide, oxaliplatin, pemetrexed, and paclitaxel (25–30). For example, anthracyclines, including doxorubicin, idarubicin and mitoxantrone, are identified as ICD inducers in the mouse models of colorectal cancer (27), and cyclophosphamide can also induce ICD as shown in glioma mouse models (30). In addition, single-agent pemetrexed or docetaxel can induce ICD in 16 NSCLC patients, with increasing plasma concentration of soluble calreticulin (31). ICD is a form of regulated cell death, which activates an adaptive immune response in immunocompetent hosts (32, 33). The hallmarks of ICD include the exposure and release of numerous damage-associated molecular patterns (DAMPs), the phosphorylation of eukaryotic translation initiation factor 2 subunit-α (eIF2α), and the activation of type I IFN signaling and autophagy. Among them, DAMPs play a vital role in stimulating adaptive immune response, which contain increasing extracellular ATP, surface-exposed calreticulin, and released high mobility group box protein 1 (HMGB1) (34, 35). Increasing extracellular ATP, which provides a ‘find me’ signal, attracts antigen-presenting cells through binding with the purinergic receptors (36, 37) and stimulates dendritic cell (DC) maturation (38–41). Moreover, ATP triggers the formation of the NOD-like receptor family, pyrin domain containing-3 protein (NLRP3)-dependent caspase-1 activation complex (42), which promotes the secretion of IL-1β and IL-18 (43), to stimulate adaptive immune response (44). In addition, the P2X7 receptor (P2X7R), is expressed on various immune cells, and its expression sensitizes cells to enhanced ambient ATP concentrations, which modulates energy metabolism and T cell growth and differentiation (45). The exposure of calreticulin from endoplasmic reticulum to cell surface depends on the rapid phosphorylation of eIF2α and ERp57 (46), and provides an ‘eat me’ signal to promote phagocytosis by DCs, provoking the adaptive immune response (27, 47). However, mutant calreticulin with loss of the KDEL sequence is secreted to extracellular space, and may inhibit the phagocytosis of dying cancer cells by DCs through saturating binding sites on DCs (48). Moreover, the interaction between calreticulin and toll-like receptor 4 (TLR4) expressed on tumor cell surface promotes the secretion of TNFα and CCL19, which facilitates the migration and maturation of DCs, to limit the tumor progression *in vivo* (49). HMGB1, released from dying cancer cells, binds with TLR4 expressed on DCs to strengthen the antigen-presenting activity of DCs through activating the PI3K/AKT/mTOR signaling pathway, and promotes anti-tumor immune response of T cells (50, 51). Different HMGB1 isoforms exert different effects on immune response, and the reduced form is responsible for the activation of DCs (52). In addition, the administration of carboplatin, cisplatin, and gemcitabine increases the PD-L1 expression on tumor cells, and shows a better efficacy when combining with ICIs in NSCLC mouse models (53–55), in which the role of cisplatin has been validated in human NSCLC tumor samples (56). Moreover, cisplatin increases the expression level of MHC class I antigen on tumor cells, and subsequently augment CTL-mediated attack to tumor cells (57, 58). In summary, chemotherapy-induced ICD promotes the cross-presentation of tumor antigen to CD8+ cytotoxic T lymphocytes (CTLs), which limits tumor progression effectively.

## Changing the proportion and activity of immune cells in TME

Chemotherapeutic agents have been validated that they can interact directly with immune cells to stimulate anticancer immunity through several mechanisms, which changes the infiltration and activity of immune cells in TME, including the depletion of immunosuppressive cells, the activation of immune effector cells, and promoting the proliferation of immune cells. Tumor-infiltrating Tregs promote tumor progression by inhibiting endogenous cytotoxic T cell responses (59), and it has been reported that some chemotherapy drugs can decrease the amount of Tregs (60–63). For example, the frequencies of intra-tumoral Tregs decreases significantly after the pretreatment of paclitaxel and cisplatin in a murine lung carcinoma model (64). Paclitaxel selectively decreases the size of Treg population in peripheral blood of patients with NSCLC, which may promote the upregulation of CD95 on Tregs, leading to cell apoptosis (65). Moreover, cyclophosphamide also decreases the amount of intra-tumoral Tregs in NSCLC mouse model (66), which is also identified in patients with recurrent prostate cancer (67). In addition, the amount of MDSCs, another immunosuppressive cell that helps tumor cells evade immune destruction (68, 69), is decreased after the administration of docetaxel, gemcitabine and 5-fluorouracil in mouse experiments (70–73). As shown in mouse models of melanoma (B16F10), gemcitabine significantly reduces the immunosuppressive state by decreasing the size of MDSCs and Tregs (70). Moreover, the number of circulating MDSCs in patients with pancreatic cancer decreases after the administration of gemcitabine, which provides precise clinical evidence (74), which is also validated in glioblastoma patients (75). The combination of gemcitabine and cisplatin reduce the amount of Tregs and regulatory B cells in in nasopharyngeal carcinoma patients (76). In addition, cyclophosphamide plus gemcitabine combination chemotherapy reduces the immunosuppressive state through decreasing the number of Tregs and MDSCs, enhancing anti-tumor immune response in colon carcinoma-bearing mice (63). In summary, the use of chemotherapeutic agents impairs the immunosuppressive role of TME by reducing the number of immunosuppressive cells, which enhances the anti-tumor activity.

In addition, several animal studies have validated that some chemotherapeutic agents, including platinum drugs and docetaxel, have the ability to promote the infiltration of CD8+ T cells, enhancing their anti-tumor effects (53, 60, 77–79). It has been reported that the administration of cisplatin and docetaxel increases intra-tumoral CD8+ T cell infiltration in a phase I/II study of neoadjuvant chemotherapy for resectable NSCLC (80). The increasing CD8+ T cell infiltration contributes to enhancing the anti-tumor activity and limits the tumor progression (81). Meanwhile, further studies explaining the mechanisms that platinum drugs increase the infiltration of CD8+ T cells have been conducted. For example, oxaliplatin enhances the secretion of CXCL9, CXCL10, and CXCL11 from tumor cells, which attracts CD8+ effector T cells through interacting with CXCR3 (82, 83), and subsequently promotes T cell infiltration in tumor tissues. In addition, neoadjuvant chemotherapy increases the infiltration of tissue resident memory T cells (TRMs) in resectable NSCLC patients, which will provide long-term anti-tumor immune response (84). TRMs express high levels of inhibitory receptors, such as PD-1 and Tim-3, and it has been validated that TRMs show a significant expansion and enhancing cytotoxic capacity after the administration of PD-1 inhibitors (85–88). Moreover, cisplatin can increase the infiltration of CD8+ T cells *via* activating cGAS-STING signaling in K-ras-driven tumor cells (77), and enhance the killing effects of CD8+ T cells through Fas/Fas ligand interactions in NSCLC mouse model (89), which may provide an inflammatory environment to enhance the anti-tumor activity of ICIs.

Chemotherapeutic agents also increase the infiltration of APCs in tumor tissues, such as DCs and macrophages, and enhances the anti-tumor immune response. Anthracycline-based chemotherapy increases the intra-tumoral infiltration of DCs in fibrosarcoma and breast cancer mouse models (90, 91). A prospective study suggests that the responsiveness of DCs recovers after anthracycline-based neoadjuvant chemotherapy in breast cancer patients (92). Except for above mentioned ATP signaling, CCL2/CCR2 axis may also be required for the intratumoral recruitment of DCs (90). In addition, platinum (IV) complexes increase the infiltration of M1 macrophages by decreasing the expression of CD47 in lung cancer mouse model, which is overexpressed on tumor cells and limits the antigen-presenting activity of APCs (93, 94). DCs play a vital role in maintaining CD8+ T cell function within tumors, and promote ICIs mediated anti-tumor immunity (95, 96). Furthermore, DCs may license PD-1 blockade *via* CD28 costimulation (97), and conventional DCs express genes correlated with CXCL9, which is related to PD-1 inhibitor response (98–100). In summary, chemotherapy-induced APCs increase in tumor tissues may contribute to ICI-enhanced anti-tumor activity.

## Immune cell metabolic reprogramming

As a crucial hallmark of cancer, metabolism reprogramming provides a favorable immunosuppressive microenvironment for the tumor progression (101–103). Chemotherapy usually influences patients’ nutritional status, and the serum of patients with lung cancer is accompanied by metabolic alterations, including glycolysis and lipid metabolism, phosphatidylcholine biosynthesis as well as amino acid metabolism (104). What’s more, metabolic reprogramming is closely involved in the activation and of T cells. For example, the activation of T cells needs higher levels of glycolysis and mitochondrial respiration (105). Thus, chemotherapy may influence the immune effects of T cells through metabolic processes. Pemetrexed has been validated to increase mitochondrial function of T cells in colon cancer mouse model, which is necessary for the activation of T cells (26, 106). Nevertheless, there is little researches about the influence of other chemotherapeutic agents on metabolic reprogramming of immune cells, and further metabolomics study is necessary to explore this influence in preclinical and clinical studies.

## Microbiome reprogramming

Gut microbiome is a complex ecosystem that regulates the interaction of the human and their environment, which has a potential impact on anti-tumor immune responses through various mechanisms (107), and is closely correlated with the efficacy of ICIs in cancer treatment (108–110). Chemotherapy has been validated to change the proportion of gut microbiome. The abundance of the Firmicutes phylum and Enterobacteriaceae increase after the administration of pemetrexed in the patient-lung-derived tumor xenograft mouse models (111). The selected species of Gram-positive bacteria are induced into the secondary lymphoid organs by cyclophosphamide, and stimulates the memory Th1 immune responses, which promotes the anti-tumor immune response (112). Therefore, the microbiome reprogramming induced by chemotherapy may provoke the anti-tumor effects of ICIs. However, the influence of chemotherapy on local microbiome needs to be investigated further, and the impacts of chemotherapy on ICI administration are required to be validated in clinical researches from the perspective of microbiome reprogramming.

## ICIs combined with chemotherapy in various cancers

Considering the limitations of ICIs monotherapy in controlling tumor progression, a large amount of studies are devoted to the safety and effectiveness of ICIs combined with standard-of-care chemotherapies. ICIs combined with chemotherapy has been approved for the treatment of certain cancer types by FDA, and more than 600 ongoing clinical trials are devoted to exploring or optimizing for various oncological indications. In patients with NSCLC, pembrolizumab plus a platinum and pemetrexed provides a better prognosis compared to pembrolizumab monotherapy (113), owing to the immune activation of these chemotherapy drugs. Similar effects are also observed in patients with untreated locally incurable recurrent or metastatic head and neck squamous cell carcinoma treated with pembrolizumab plus a platinum and 5-fluorouracil (114, 115). In addition, atezolizumab plus carboplatin and nab-paclitaxel prolongs the overall survival and progression-free survival in patients with advanced non-squamous NSCLC (116). Neoadjuvant carboplatin and paclitaxel chemotherapy increases the amount of central memory CD8+ T cell in peripheral blood of patients with advanced serous ovarian cancer, enhancing antigen processing and presentation (117). However, the further studies are needed to investigate the corresponding mechanisms of neoadjuvant carboplatin and paclitaxel chemotherapy in inhibiting NSCLC progression. Although ICIs combined with chemotherapy significantly prolongs the survival of patients with tumors, it is necessary for us to evaluate the toxicity of the combination therapies. ICIs combined with chemotherapy may cause hematological, gastrointestinal, and renal toxicity, and contribute to hypothyroidism, hyperthyroidism, pneumonitis, hepatitis, severe skin reactions, colitis, and infusion reactions (118–121). However, the current understanding of adverse reactions of combined therapies is incomplete, and it is necessary to describe the preferable adverse reactions of different combinations, which is beneficial for balancing the safety and efficiency of the corresponding treatment.

## Discussion

ICIs have made great contributions to the survival of patients with cancers. However, the low response rate of patients to ICIs prompts us to explore the possibility of ICIs combination with other therapies. As a routine therapy, chemotherapy attracts much attention because of its immune stimulation activity, and ICIs combination with chemotherapy achieves great effects in clinical trials. FDA has approved several combination therapies for the treatment of advanced NSCLC in the first-line setting (122). To clarify the mechanism how chemotherapy promotes curative effect of ICIs is beneficial for the application of the combination therapies. As mentioned above, chemotherapeutic agents-induced ICD and their immune stimulation activity are considered as the main mechanism of combination therapy. However, the impacts of chemotherapy are so complex that chemotherapy may influence ICI-mediated anti-tumor responses through various routes.

With the development of sequencing technology, the landscape of immune cells within tumor tissues has been gradually revealed, and more and more cellular components have been recognized. For example, B cells represent a vital component of infiltrating immune cells in a variety of solid tumors, and play a dual role in modulating anti-tumor immune response (123). Chemotherapy reduces the amount of adenosine-producing B cells, which may reduce potential immunosuppression in TME (124). However, there is little research about the influence of chemotherapy on B cell subpopulations and their activity.

Moreover, cancer-associated fibroblasts (CAFs) account for more than 50% of stroma cells in TME, and various CAF populations have been identified based on the results of single-cell sequencing analyses, including the cancer-associated myofibroblasts (myCAFs), inflammatory-like CAFs (iCAFs), and the antigen-presenting CAFs (apCAFs) (125). CAFs are involved in regulating tumor immune response and the efficacy of immunotherapy through several routes (126). For example, CAFs increase the ratio of FoxP3+ (Tregs) and CD8+ tumor-infiltrating lymphocytes *via* IL-6 in TME, and IL-6 blockade enhances the immunotherapy efficacy in esophageal cancer models (127). Several studies have reported that CAFs protect tumor cells from apoptosis induced by chemotherapy, while insulin-like growth factors secreted by CAFs enhanced the anti-tumor effects of osimertinib in mice model (125, 128). Moreover, CAFs can activate the NLRP3 inflammasome through sensing DAMPs in breast cancer, which leads to a pro-inflammatory signaling (129). The influence of chemotherapy on diverse CAFs and their association with the immunotherapy efficacy has not been explored comprehensively, and further researches are needed.

Except for the influence of chemotherapeutic agents on novel cellular components in TME, chemotherapy-induced microbiome reprogramming also contributes to the combination of ICIs and chemotherapy. Although the impacts of chemotherapy on gut microbiome have been explained partly in animal experiments, the overall landscape of gut microbiome reprogramming after treating with different chemotherapeutic agents needs to be described in more preclinical and clinical researches. Beyond that, the influence of chemotherapy on local microbiome also needs to be investigated further, and the impacts of chemotherapy on ICI administration are required to be validated in clinical researches from the perspective of microbiome reprogramming.

In addition, the administration approaches of ICIs combination with chemotherapy are necessary to be improved. Firstly, a proper combination therapy can maximize the clinical benefit and minimize the adverse drug reactions. As mentioned above, different chemotherapy drugs stimulate effective anti-tumor immune responses through different mechanisms, and cancer patients may reap more benefits with appropriate combination therapies after evaluating the tumor conditions, including PD-L1 expression level, immune cell infiltration, and tumor mutation burden. However, there is little clinical trials about comparing the effectiveness of combination therapies of different chemotherapeutic drugs and ICIs. Secondly, chemotherapeutic regimens and ICIs are administrated simultaneously in most clinical trials. Nevertheless, it has reported that the sequence of administrating chemotherapeutic agents and ICIs has an impact on the efficiency of the combined therapies in several animal experiments. The administration of anti‐CTLA‐4 antibody after injecting cyclophosphamide significantly inhibits the tumor progression in the CT26 colon carcinoma model, while the reverse administration sequence leads to the apoptosis of anti-tumor CD8+ T cells (130). Apart from this, the time interval from chemotherapy to immunotherapy may also influence the response to ICIs. In the long rest period group, the frequency of the Th1 subset and PD-1 + CD8+ T cells are significantly higher than that in the short rest period group, which provides a novel perspective for the application of combination therapies (131). However, there is no more research about appropriate sequence and proper time interval, and it needs more researches to study the influence of time interval on anti-tumor effects. Lastly, proper dose may be the most important respect to effectively stimulate the maximum anti-tumor immune response and minimize adverse reactions. For example, low dose cisplatin and oxaliplatin increase the number of circulating CD4+ and CD8+ T cells, while high dose regimens decrease the size of lymphocyte in a mouse model of colon cancer (132). Metronomic low dose cyclophosphamide enhance anti-tumor immune response by selectively reducing the amount of Treg cells in tumor patients (133), while high dose cyclophosphamide completely eradicates the hemopoietic cell (134). In addition, a novel administration mode called medium-dose intermittent chemotherapy provokes a striking response depending on the activation of a sustaining anti-tumor immune response (66, 135). Therefore, appropriate dosage for combined therapies is necessary to be investigated in further studies.

In summary, ICIs combination with chemotherapy has shown a better anti-tumor response and provides a more beneficial survival, compared to ICIs monotherapy. This benefit is supported by a strong cancer biology rationale, which induce a better immune response. Hence, the evaluation of the panoramic dynamic immune landscape of TME will be helpful to understand tumor pathogenesis and provide novel approaches for cancer treatment.

## Author contributions

All authors have made a direct contribution to the work, and approved it for publication.
